# Prediction of recovery from multiple organ dysfunction syndrome in pediatric sepsis patients

**DOI:** 10.1093/bioinformatics/btac229

**Published:** 2022-06-27

**Authors:** Bowen Fan, Juliane Klatt, Michael M Moor, Latasha A Daniels, Swiss Pediatric Sepsis Study, Philipp K A Agyeman, Christoph Berger, Eric Giannoni, Martin Stocker, Klara M Posfay-Barbe, Ulrich Heininger, Sara Bernhard-Stirnemann, Anita Niederer-Loher, Christian R Kahlert, Giancarlo Natalucci, Christa Relly, Thomas Riedel, Christoph Aebi, Luregn J Schlapbach, Lazaro N Sanchez-Pinto, Philipp K A Agyeman, Luregn J Schlapbach, Karsten M Borgwardt

**Affiliations:** Department of Biosystems Science and Engineering, ETH Zurich, Basel 4058, Switzerland; SIB Swiss Institute of Bioinformatics, Lausanne 1015, Switzerland; Department of Biosystems Science and Engineering, ETH Zurich, Basel 4058, Switzerland; SIB Swiss Institute of Bioinformatics, Lausanne 1015, Switzerland; Department of Biosystems Science and Engineering, ETH Zurich, Basel 4058, Switzerland; SIB Swiss Institute of Bioinformatics, Lausanne 1015, Switzerland; Division of Critical Care, Ann and Robert H. Lurie Children’s Hospital of Chicago, Chicago, IL, USA; Division of Critical Care, Ann and Robert H. Lurie Children’s Hospital of Chicago, Chicago, IL, USA; Department of Pediatrics, Inselspital, Bern University Hospital University of Bern, Bern 3010, Switzerland; Department of Intensive Care and Neonatology, and Children’s Research Center, University Children’s Hospital Zurich, Zurich 8032, Switzerland; Paediatric Intensive Care Unit, Child Health Research Center, Queensland Children’s Hospital, The University of Queensland, Brisbane, Australia; Department of Biosystems Science and Engineering, ETH Zurich, Basel 4058, Switzerland; SIB Swiss Institute of Bioinformatics, Lausanne 1015, Switzerland

## Abstract

**Motivation:**

Sepsis is a leading cause of death and disability in children globally, accounting for ∼3 million childhood deaths per year. In pediatric sepsis patients, the multiple organ dysfunction syndrome (MODS) is considered a significant risk factor for adverse clinical outcomes characterized by high mortality and morbidity in the pediatric intensive care unit. The recent rapidly growing availability of electronic health records (EHRs) has allowed researchers to vastly develop data-driven approaches like machine learning in healthcare and achieved great successes. However, effective machine learning models which could make the accurate early prediction of the recovery in pediatric sepsis patients from MODS to a mild state and thus assist the clinicians in the decision-making process is still lacking.

**Results:**

This study develops a machine learning-based approach to predict the recovery from MODS to zero or single organ dysfunction by 1 week in advance in the Swiss Pediatric Sepsis Study cohort of children with blood-culture confirmed bacteremia. Our model achieves internal validation performance on the SPSS cohort with an area under the receiver operating characteristic (AUROC) of 79.1% and area under the precision-recall curve (AUPRC) of 73.6%, and it was also externally validated on another pediatric sepsis patients cohort collected in the USA, yielding an AUROC of 76.4% and AUPRC of 72.4%. These results indicate that our model has the potential to be included into the EHRs system and contribute to patient assessment and triage in pediatric sepsis patient care.

**Availability and implementation:**

Code available at https://github.com/BorgwardtLab/MODS-recovery. The data underlying this article is not publicly available for the privacy of individuals that participated in the study.

**Supplementary information:**

Supplementary data are available at *Bioinformatics* online.

## 1 Introduction

Sepsis is a life-threatening disease, which is defined as dysregulated host response to infection and the cause of millions of deaths all over the world every year (Rudd *et al.*, 2020; Singer *et al.*, 2016). Sepsis disproportionally affects children as it occurs 22 times per 100 000 person-year in children, which translates to ∼1.2 million cases per year (de Souza and Machado, 2019). Even in high-income countries with low childhood mortality, sepsis and invasive infections contribute to 10–20% of childhood deaths, and account for one out of four deaths in the pediatric intensive care unit (PICU; Ladhani *et al.*, 2010; Lantto *et al.*, 2013). Moreover, children who survive sepsis may suffer from both short- and long-term consequences, resulting in a lifelong burden to patients, families and society (Giannoni *et al.*, 2018; Schlapbach *et al.*, 2011).

Multiple organ dysfunction syndrome (MODS), defined as two or more concurrent organ systems dysfunctions, has been associated with increased mortality and constitutes a major cause of death in the PICU (Lin *et al.*, 2017). The pathophysiology of MODS is characterized by a severe, systemic, uncontrolled inflammatory process that leads to multiple organ system dysfunctions. A patient who exhibits MODS at the time of sepsis onset is much less likely to recover through treatment than a patient without MODS. For those already in MODS when acquiring sepsis, it is crucial to investigate their chances to recover to a mild state with zero or single organ dysfunction (Z/SOD), thus providing the clinicians the opportunity to take extra care and enable timely interventions for those who may otherwise not be likely to recover.

Recently, the development of supervised machine learning models using electronic health records (EHRs) has achieved tremendous progress in patient outcome prediction tasks in different clinical settings, such as pathology, radiology and intensive care (Komorowski *et al.*, 2018; Lakhani and Sundaram, 2017; Meyer *et al.*, 2018). Traditional, rule-based methods for patient outcome prediction have certain limitations, such as low flexibility, a lack of personalization and excessive false-positive rates (Banda *et al.*, 2018; Gehrmann *et al.*, 2018). Data-driven machine learning models can help overcome these limitations, thereby potentially yield higher performance and provide efficiency, reliability and interpretability if designed suitably. Moreover, machine learning has also proven its superiority in the early prediction of sepsis onset and development of MODS (Bose *et al.*, 2021; Moor *et al.*, 2021a,b), but early prediction of the recovery from MODS in pediatric sepsis patients has not yet been attempted by that. To fill this gap, we here developed a proof-of-concept machine learning-based approach for the early prediction of MODS recovery with a time horizon of 7 days. We also assessed the generalizability of the model by evaluating its predictive performance on a second pediatric sepsis cohort from another clinical institution. Our goal in this work is to show that by incorporating the physiological measurements and additional information of the patients, the machine learning model can deliver an accurate prediction of the likelihood of MODS recovery within a week and has a strong ability to generalize to another clinical setting.

## 2 Data

### 2.1 Swiss Pediatric Sepsis Study

The Swiss Pediatric Sepsis Study (SPSS) is a prospective, national, observational, multi-center cohort study investigating blood culture-proven sepsis in children younger than 17 years old recruited from September 1, 2011 until December 31, 2016. This study involves 10 major pediatric hospitals in Switzerland, which accounts for ∼98% of all PICU admissions and 78% of all hospital admissions in Switzerland, with an International Classification of Diseases (ICD)-10 code for pathogen-specific sepsis. SPSS aimed to advance the understanding of individual susceptibility to sepsis in children, as well as to contribute to the identification of novel targets for personalized medicine. Inclusion and exclusion criteria have been detailed in Agyeman et al. (2017). In brief, all children younger than 17 years with blood culture-proven invasive bacterial or fungal infection were included if they met systemic inflammatory response syndrome criteria—according to 2005 pediatric consensus definitions (Goldstein *et al.*, 2005)—at the time of blood culture sampling and the pathogen isolated was not considered a contaminant.

The SPSS dataset includes the daily number of dysfunctioning organ systems (cardiovascular, respiratory, hepatic, renal, neurological and hematological) according to the 2005 consensus definitions (Goldstein *et al.*, 2005). In patients with at least one organ dysfunction, daily values are available as long as an organ dysfunction was present up to 6 days after blood culture sampling, which we employed to calculate the outcome label of whether a patient with MODS on the blood culture sampling day (denoted as *d*_0_) would recover to Z/SOD after 6 days (denoted as *d*_6_). All organ dysfunction scores are based on laboratory, physiological or clinical data captured in the database, and any missing value for the determination of organ dysfunction was set to normal. MODS is then defined as the presence of organ dysfunction in at least two different organ systems according to 2005 consensus definitions. The recovery from the state of MODS in the first week since sepsis onset is of high clinical relevance and has been previously studied already (Sanchez-Pinto *et al.*, 2020). In total, the dataset includes 4498 daily records of 1247 7-day episodes from 1149 patients (an individual patient can have more than one sepsis episode and different episodes from the same patient were treated independently). The MODS recovery prediction model was developed based on the clinical records from the blood culture sampling day *d*_0_. Out of the 1247 episodes, 991 were removed as they corresponded to patients who did not exhibit MODS or no record was available on *d*_0_, and hence no label could be assigned. Out of the remaining 256 episodes (from 240 patients), 50 deceased within 7 days after *d*_0_, and 89 still suffered from MODS on *d*_6_. These two groups add up to 138 episodes, and they were assigned to the negative class (no recovery). The rest 118 recovered to a relatively mild state (Z/SOD) on *d*_6_ and were assigned to the positive class.

The SPSS dataset further comprises 22 physiology features (including vital signs such as heart rate, respiratory rate, systolic blood pressure, temperature; markers of inflammation such as white blood cell count, neutrophil count, lymphocyte count; markers of organ dysfunction or injury such as alanine aminotransferase, total bilirubin, creatinine), 17 organ failure scores (including the pediatric logistic organ dysfunction (PELOD-2) score (Leteurtre *et al.*, 2013) and the pediatric sequential organ failure assessment score (Matics and Sanchez-Pinto, 2017), 16 indicators of complex chronic condition (including chronic neurological disorder, malformation or genetic disorder and dependence on technological assistance (Feudtner *et al.*, 2014), 3 patient demographic features (including age, ethnicity and sex), 34 infection characteristic indicators (including the site of infection and pathogen group). For physiology variables and scores, the dataset includes the most abnormal value per day for 7 consecutive days starting and including when the blood culture was taken (*d*_0_). Supplementary Table S1 presents a detailed overview of the variables contained in the SPSS dataset, as well as the percentage of missing values per variable.

For the complete demographic information and clinical characteristics of the 256 episodes with MODS on blood culture sampling day and included in this analysis, see Supplementary Table S2.

**Fig. 1.**
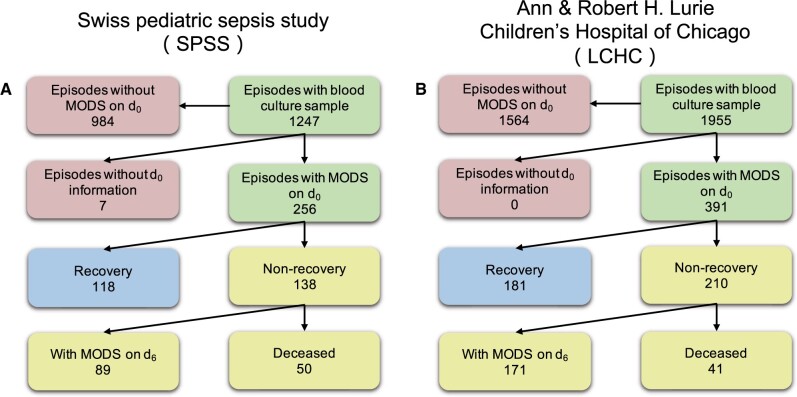
Tree diagram of prediction cohort selection in both databases. (**A**) From the overall 1247 episodes in the SPSS cohort, seven of them who do not have any information available were excluded. Further, 984 episodes were removed as they corresponded to patients who were not in MODS already on *d*_0_, and hence no outcome could be assigned. The remaining 256 episodes constitute the set of instances used for the MODS recovery prediction task. Out of these 256 episodes, 138 episodes belong to the negative class (of which 50 died within 7 days and 89 still exhibited MODS on *d*_6_, with one of them belonging to both cases), while the rest 118 episodes belong to the positive class (recovered to Z/SOD on *d*_6_). (**B**) Out of 1955 episodes in the LCHC database, 1564 episodes without MODS on *d*_0_ were excluded. The remaining 391 episodes constitute the external validation set, with 181 episodes recovered on *d*_6_, whereas the rest 210 episodes did not recover

**External validation set:** The dataset used to externally validate the MODS recovery model developed on the SPSS cohort is a observational retrospective dataset, which includes patients with a bacteremia younger than 17 years old who were admitted to Ann & Robert H. Lurie Children’s Hospital of Chicago (LCHC) between July 1, 2012 and July 1, 2021. The same variables as in SPSS were extracted from the EHRs in the LCHC dataset and the same MODS definition was applied. The patient-inclusion flow charts of both datasets are presented in Figure 1.

A total number of 44 features were used for the development of the machine learning models, which includes vital signs, laboratory tests, demographic information, clinical scores and chronic disorder conditions of the patients collected on the blood culture sampling day. Table 1 presents an overview of the extracted information from the two pediatric sepsis databases.

**Table 1.** Information extracted from the two databases involved in this study

Types	Contents
Vital signs	Heart rate (highest), respiratory rate (highest), mean arterial pressure (lowest), systolic blood pressure (lowest), temperature (highest/lowest) and oxygen saturation (lowest)
Laboratory tests	Lactate (highest), inspired oxygen fraction (highest), platelet count (lowest), white blood cell count (highest/lowest), lymphocyte cell count (lowest), total neutrophil cell count (lowest), total bilirubin (highest), serum creatinine (highest), alanine aminotransferase (highest), INR (International Normalized Ratio) coagulation (highest), partial arterial CO_2_ pressure (highest), partial arterial O_2_ pressure (lowest) and central capillary refill time (lowest)
Clinical scores	Organ failure scores (cardiovascular, respiratory, hepatic, renal, neurological, and hematological) and Glasgow Coma Score (lowest)
Chronic condition information	Chronic disorder (neurological, cardiac, lung, urogenital, gastrointestinal, hematological or immunological, metabolic, malformation or genetic, malignancy, neonatal, surgical), dependence on technological assistance, history of transplant and total number of underlying chronic disorders
Demograhpics	Age and sex

*Notes*: The most abnormal value of each measurement was extracted within every 24 h (indicated by highest and lowest). Except for the chronic condition indicators and sex being binary variables here, the rest features are numeric.

## 3 Materials and methods

The investigation presented in this work was designed as a retrospective analysis of pediatric patients from two different clinical cohorts with blood-culture confirmed bacteremia and MODS using both machine learning and statistical methods.

### 3.1 Prediction models

We implemented and compared several classification models for the MODS recovery prediction task, including logistic regression (LR), random forest (RF), support vector machines (SVM), multi-layer perceptrons (MLP) and light gradient boosting machines (lightGBM; Ke *et al.*, 2017). All models were optimized to minimize the binary cross-entropy loss between the 7-day recovery labels and the prediction scores generated by the machine learning model. For each type of model, we ran a randomized hyper-parameter search with 50 iterations. As for a clinical baseline, we investigated how well the recovery from MODS could be predicted with the PELOD-2 scores together with age and sex of the patients, which have been shown to predict mortality and organ dysfunction in large databases and at present remain the best-validated criteria (Schlapbach *et al.*, 2022; Weiss *et al.*, 2022).

With respect to data pre-processing, we first imputed the missing fraction of inspired oxygen as 21% (i.e. atmospheric condition), and all numerical features in the raw data were standardized to zero mean and unit variance, and the remaining missing values were imputed with zero. Then for the internal validation, we employed a nested cross-validation (CV) scheme owing to the relatively small number of patients available, and each run in the CV we randomly split the data into a training (60%), validation (20%) and test (20%) set, while all three of them were stratified to preserve the specific positive label prevalence in the dataset. We repeated this split for 10 independent runs, and in each run, we used the training and validation sets for the hyper-parameter optimization. All machine learning models were internally evaluated on the SPSS cohort by the mean of the area under the receiver operating characteristic (AUROC) and the area under the precision-recall curve (AUPRC) from the 10 test sets. For the external validation, we selected the best-performing model based on the mean AUROC of the internal test sets and retrained the model on the entire dataset and validated it on the other. All performances were reported in AUROC and AUPRC.

To further examine the predictive performance of the model for the different time horizons from 1 to 6 days after sepsis onset, we evaluated relative AUPRC (AUPRC divided by positive class prevalence) on both SPSS and LCHC data from *d*_1_ to *d*_6_ with the SPSS-trained RF models. We adopted the evaluation strategy for *d*_6_ for all other days.

### 3.2 Model interpretation and analysis

In order to improve the clinical significance of the model and also to identify which features are most relevant to the predictions made by it and thereby determine risk factors among patient characteristics—be it in terms of the physiological state a patient is in or permanent attributes such as demographics and complex chronic conditions, we calculated SHAP (SHapley Additive exPlanations) values (Lundberg and Lee, 2017), which can interpret the predictions of various kinds of black-box machine learning models. The main idea of SHAP values is that when explaining each feature in the instance, the model is evaluated using all possible sets of features with and without the target feature (Elshawi *et al.*, 2019).

We further examined if there exists any enrichment of certain pathogen group, age group or site of infection in the wrongly classified instances (false positive and false negative) compared with their true class (ground truth negative and ground truth positive). This analysis aims to investigate the wrong decisions made by the model and thus better interpret the model itself. The enrichment was tested by means of comparing the actual fraction of, e.g., a pathogen group in the false positives to the distribution of that fraction in randomly sampled instances from the ground truth negatives. This analysis was conducted with the threshold of recall fixed at 80%. Statistical significance of observed enrichment was then determined at a 5% significance level while employing a Bonferroni correction to account for the multiple testing problem when testing each characteristic group of interest.

## 4 Results

### 4.1 Patient characteristics

These 256 selected episodes from SPSS have a median age at sepsis onset of 6.58 months (IQR (Interquartile Range) 0.30–53.54). 106 (41.4%) 7-day episodes occurred in neonates, 43 (16.8%) in previously healthy children, and 107 (41.8%) in children with comorbidity. Fifty children deceased in the first 7 days after sepsis onset with a median time to death of 1 day (IQR 0–2). We compared the main characteristics between recovery and non-recovery samples from the SPSS and LCHC cohorts in Table 2.

**Table 2.** Characteristics of patients from SPSS and LCHC databases

Variable	Recovery (SPSS)	Non-recovery (SPSS)	Recovery (LCHC)	Non-recovery (LCHC)
*n*	118	138	181	210
Age (months)	16.6 (0.48–85.1)	1.75 (0.27–33.72)	42 (4.6–106.8)	7.7 (0.4–59.6)
Female	53 (55.1%)	45 (32.6%)	75 (41.4%)	88 (41.9%)
Male	65 (44.9%)	93 (67.4%)	106 (58.6%)	122 (58.1%)
Number of organ failure on *d*_0_	2 (2–3)	3 (2–4)	3 (2–4)	2 (2–3)
Death within 7 days	0	50	0	41
MODS on *d*_6_	0	89	0	171
Ethnicity				
White European	94 (79.7%)	94 (68.1%)	84 (46.4%)	81 (38.6%)
Asian	3 (2.5%)	4 (2.9%)	10 (5.5%)	16 (7.6%)
African	6 (5.1%)	13 (9.4%)	35 (19.3%)	49 (23.3%)
Other	15 (12.7%)	27 (19.6%)	52 (28.7%)	64 (30.5%)
Risk category				
Neonate	51 (43.2%)	65 (47.1%)	24 (13.3%)	57 (27.1%)
With comorbidity	41 (34.8%)	56 (40.6%)	174 (96.1%)	209 (99.5%)
Previously healthy	26 (22.0%)	17 (12.3%)	7 (3.9%)	1 (0.5%)
PICU Admission				
Yes	93 (78.8%)	133 (96.4%)	104 (57.5%)	115 (54.8%)
No	25 (21.2%)	5 (3.6%)	77 (42.5%)	95 (45.2%)

### 4.2 Model performance

**Fig. 2.**
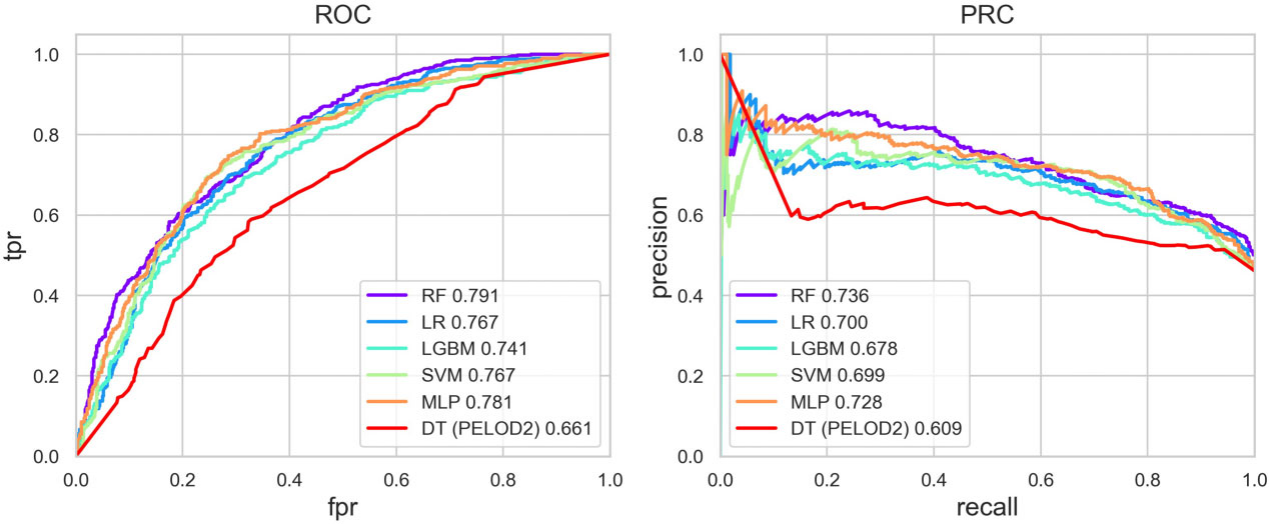
Comparison plots of the performances of five classification models on the SPSS cohort. This shows the ROC and PRC generated from 10-fold CV on the SPSS cohort. The mean values over the test sets are presented. fpr, false positive rate; tpr, true positive rate; RF, random forest; LR, logistic regression; SVM, support vector machine; LGBM, light gradient boosting machine; MLP, multi-layer perceptron; DT (PELOD2), clinical baseline model based on a decision tree developed with PELOD2 scores and age and sex of the patients

**Fig. 3.**
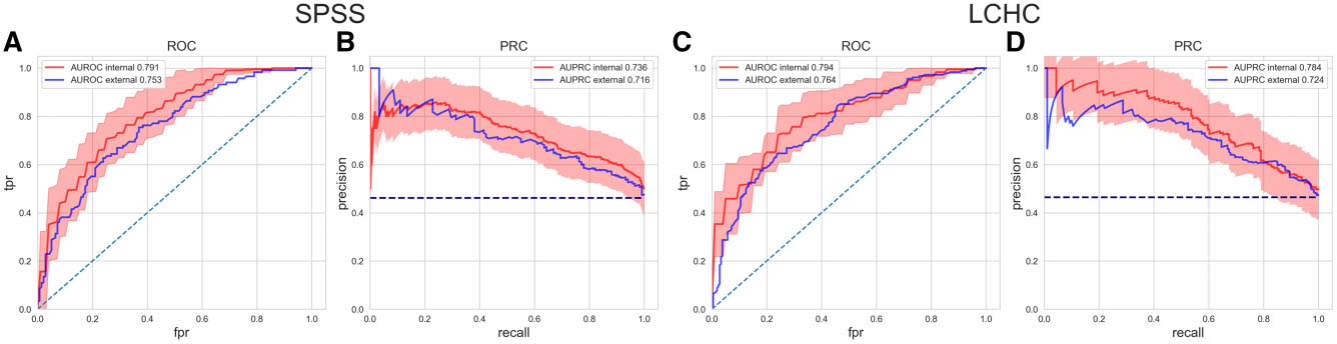
Internal and external validation performance. This shows the ROC and PRC from the SPSS data and LCHC data, respectively. Curves with uncertainty band are from 5-fold cross internal validations, and the shaded area represents the prediction uncertainty. Curves without uncertainty are from external validations. (**A** and **B**) Internal and external validation performance on the SPSS data. (**C** and **D**) Internal and external validation performance on the LCHC data

As mentioned earlier, we implemented and compared five different machine learning models for the MODS recovery prediction. From Figure 2, we can see the best-performing (evaluated by AUROC) MODS recovery model was acquired using an RF, with a mean AUROC of 79.1% (SD 5.7%) and AUPRC of 73.6% (SD 7.9%, with a positive class prevalence of 46.1%) on the 10 test sets of the CV scheme. For completeness of the study, we also did internal CV on the LCHC data with an RF model using the same features, which achieved a mean AUROC of 79.4% and AUPRC of 78.4% (see the red curves in Fig. 3).

In a second analysis, we performed the external validation. The SPSS-trained RF model achieved an AUROC of 76.4% and AUPRC of 72.4% on the LCHC data (with a positive class prevalence of 46.2%), and the LCHC-trained RF model achieved an AUROC of 75.3% and AUPRC of 71.6% on the SPSS data (see the blue curves in Fig. 3). The high performance of the model on the unseen patient cohort from another clinical institution proves that the machine learning-based approach we developed in this study generalizes well in terms of the MODS recovery prediction task.

At an operating points of 80% recall, where four out of five recovery events are correctly predicted, the prediction model (RF) reached a precision score of 63.4% internally and 60.7% externally. The evaluation was done on all the instances from the test sets (internally) and LCHC dataset (externally), with the prediction threshold of the model fixed at a point where the recall score reached 80%. Which means that for every five positive predictions made, three of them are true indicators of patients recovering from MODS state. We further evaluated the specificity, F1 score, negative predictive value (NPV) and accuracy at this threshold (see Table 3).

**Table 3.** Performance of MODS recovery prediction model at 80% recall

Model	RF	SVM	LR	MLP	lightGBM	RF on LCHC
Precision	0.634	0.625	0.633	**0.666**	0.600	0.607
Specificity	0.604	0.589	0.602	**0.655**	0.543	0.550
F1 score	0.707	0.702	0.707	**0.727**	0.681	0.690
NPV	0.779	0.775	0.778	**0.793**	0.760	0.762
Accuracy	0.694	0.687	0.693	**0.722**	0.622	0.667

*Notes:* The MLP classifier stands out when the models were evaluated at a high recall region. The text highlighted in bold indicates the highest values in the internal evaluation on the SPSS cohort. But for the same evaluation on the LCHC cohort, we only report the performance of the RF model as the selection criterion was based on the mean AUROC over all test sets of the SPSS cohort.

**Fig. 4.**
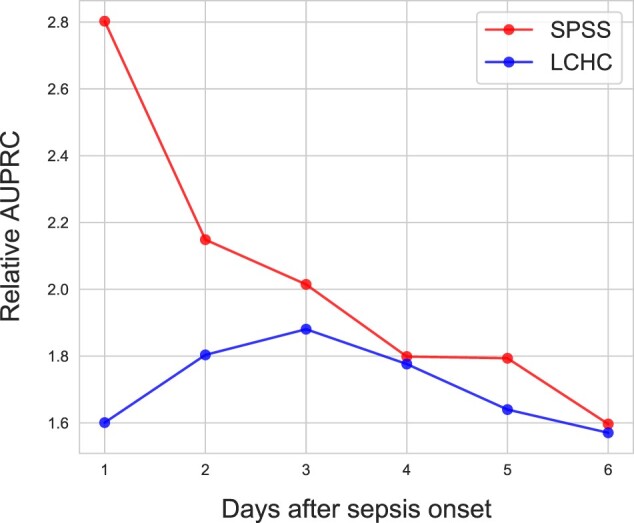
Performance versus earliness. This depicts the relative AUPRC for the MODS recovery prediction from *d*_1_ to *d*_6_ on both datasets, obtained by the SPSS-trained RF models. The numbers represent the mean relative AUPRCs on the curve of SPSS

We also evaluated if the RF model could predict the MODS recovery earlier than 6 days after sepsis onset. Figure 4 depicts the performance on both cohorts obtained by SPSS-trained RF models. The relative AUPRC on the SPSS cohort goes down when increasing the horizon. For the external validation on LCHC, the relative AUPRC increases in the first 3 days and starts to drop afterwards. On *d*_6_, which is our main focus, both internal and external validations achieve very similar performance.

### 4.3 Model interpretation

**Fig. 5.**
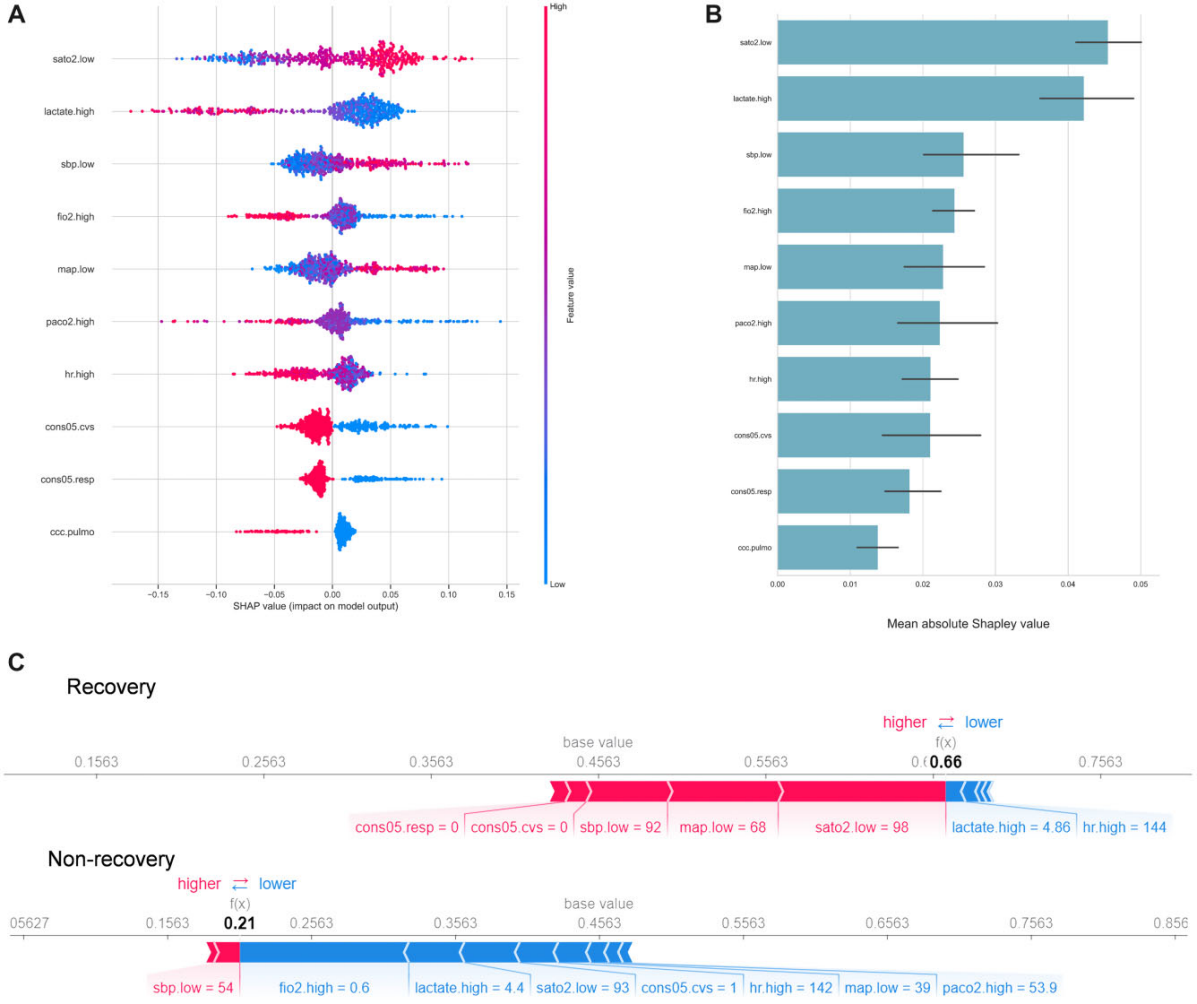
SHAP values with respect to the prediction task. (**A**) Bee-swarm plot showing SHAP values distributions for all instances in the test sets. Input feature values associated with a single prediction are color-coded, with blue/red corresponding to low/high values. The *x*-axis measures SHAP values, representing the impact of a variable with respect to the single predictions regarding the chance for a MODS recovery. (**B**) Mean absolute SHAP values over all test sets. The top 10 most impactful variables are displayed, and higher values suggests larger contributions to the model’s prediction (i.e. the prediction of MODS recovery). (**C**) Two exemplified patients, with one for each class. In these two plots, the base value indicates the average of all output values of the model, the blue/red arrows show the corresponding features ‘pushed’ the model to give to higher/lower prediction score, and the width of the arrow represents the importance of the feature (A color version of this figure appears in the online version of this article.)

In clinical practice, the interpretability of machine learning-issued decision support is highly desirable. We, therefore, calculated SHAP values (Lundberg and Lee, 2017) of physiological input variables with respect to the prediction task in order to identify those aspects of a patient’s physiological state, which are most critical for his or her risk to exhibit a 7-day MODS recovery. The SHAP values were calculated with the internally best-performing model (RF). Figure 5A depicts the bee-swarm plots, i.e. how the top features in the dataset impact the model’s output. Each instance is represented by a single dot on each row and position on the *x*-axis is determined by the SHAP value of that feature, in which the red dot represents high feature value and the blue dot represents low feature value. Figure 5B shows the mean of absolute SHAP values averaged over all 10 test sets, with the error bars indicating SD.

We found the top 10 critical variables for the MODS recovery prediction task to be: lowest oxygen saturation, the highest lactate, the lowest systolic blood pressure, the highest inspired oxygen fraction, the lowest mean arterial blood pressure, the highest partial arterial CO_2_ pressure, the highest heart rate, the cardiovascular organ failure indicator, the respiratory organ failure indicator and the chronic lung disease indicator. In Figure 5C, we provide two examples: one recovery example and one non-recovery example, to further illustrate the model’s interpretability. The patient in the first example had a relatively high heart rate and lactate level, but did not have respiratory or cardiovascular failure and his/her oxygen saturation and blood pressure were at a relatively normal level, so he or she eventually recovered. The second patient had similar oxygen saturation, lactate and heart rate level but did not recover due to a high inspired oxygen fraction (which indicates mechanical ventilation applied), low blood pressure and also a cardiovascular failure.

### 4.4 Enrichment test

When comparing the fractions of the pathogen groups, age groups and site of infections in the false positives/negatives to those in the ground truth negatives/positives, several groups of enrichment were tested to be statistically significant, see details in Table 4. In the false positives, we observed eight groups of enrichment (three types of pathogens, three age groups and two sites of infection) with an average (SD) increase of 86.0% (35.2%) compared with the ground truth negatives, and the most significant enrichment occurred in the patient with *Haemophilus influenzae* detected in blood culture (145.5%). In the false negatives, we observed five groups of enrichment (two types of pathogens, two age groups and one site of infection) with an average (SD) increase of 62.6% (28.8%), and the largest enrichment occurred in the patient with *Klebsiella* spp. detected in blood culture (108.0%).

**Table 4.** Statistically significant (at 5% level) enrichment of type of pathogen, age group and site of infection found in the FNs/FPs of the prediction from the RF model compared with the positives/negatives in the SPSS cohort

Group	From	To	%	From	To	%
Pathogen		Negatives →FPs			Positives →FNs	
Coagulase-negative staphylococci				0.206	0.323	56.8
*Klebsiella* spp				0.075	0.156	**108.0**
*Haemophilus influenzae*	0.011	0.027	**145.5**			
*Streptococcus pneumoniae*	0.098	0.153	56.1			
Group A streptococcus	0.038	0.068	78.9			
Age group		Negatives →FPs			Positives →FNs	
10–16 years	0.080	0.140	75.0			
5–9 years	0.166	0.257	54.8			
1–4 years	0.064	0.104	62.5			
<12 months				0.156	0.250	60.3
Preterm neonate				0.312	0.500	60.3
Site of infection		Negatives →FPs			Positives →FNs	
Central line-associated bloodstream				0.440	0.562	27.7
Toxic shock syndrome	0.038	0.068	78.9			
Endocarditis	0.025	0.059	136.0			

*Notes*: The analysis was done with a prediction threshold leading to 80% recall. Text highlighted in bold indicates the largest enrichment in each scenario.

FP, false positives; FNs, false negatives.

## 5 Discussion

We developed and validated a generalizable and interpretable machine learning-based approach for the early prediction of MODS recovery in pediatric sepsis patients based on two pediatric sepsis datasets from Switzerland and the USA. The approach achieved satisfactory performance in predicting the recovery from MODS to zero or single organ failure 7 days in advance in children with blood culture-proven sepsis, which can assist clinicians in identifying patients with high risk for a complicated clinical course in advance. In general, the contributions accomplished in our work can be summarized into the following parts: (i) we employed advanced machine learning model to predict the chance of recovery in pediatric patients who exhibit MODS related to sepsis; (ii) we conducted comprehensive experiments to demonstrate the proposed model can predict MODS recovery with high accuracy and also assessed its transferability to unseen patients from a different clinical site; (iii) we also used SHAP values to explain which factors are critical to the recovery process, which were discovered by the model; (iv) finally, we examined if any pathogen, site of infection or age group was enriched or depleted in the predicted positives compared with ground truth positives, to better interpret the model’s predictions.

A recent review of machine learning approaches in adult sepsis prediction shows that the majority (86%) of the studies only examined their model by CV limited to a single database (Moor *et al.*, 2021a,b), the performances in other datasets were not verified even though the models performed well in their settings. Also, in a recent large-scale study for validation of a sepsis prediction model with widespread use, the results showed the model has poor discrimination and calibration in predicting sepsis onset at the hospitalization level (Wong *et al.*, 2021). With pediatric sepsis and its related phenotypes being much less frequently studied, even fewer research works have ever focused on the generalizability in this field. The method proposed in this study was evaluated in multi-center pediatric sepsis patient databases, which allowed us to both internally and externally evaluate its predictive ability comprehensively. To the best of our knowledge, this is the first study to leverage a machine learning-based approach to predict pediatric sepsis-related outcomes and achieved a successful external validation result, even in an inter-continental setup.

It is worth noting that the prediction of MODS recovery is not redundant to mortality prediction in patients with MODS, as we found the 30-day mortality in the SPSS cohort to be 48.5% in the patients who did not recover within 7 days (67 death in 138 episodes) compared with 26.9% in all episodes (69 death in 256 episodes). This shows the clinician’s knowledge of the presence of organ dysfunction is relevant for immediate clinical management, but it is an imprecise means of predicting the final outcome (i.e. mortality). Here we, instead, proposed the MODS recovery prediction method could support patient assessment and triaging on *d*_0_.

In the evaluation within the SPSS cohort, we noticed that the five models implemented have relatively close performances. Although the RF performs the best in terms of the mean AUROC, all classification models achieved comparable and appreciable results, and outperformed the clinical baseline model by a large margin, showing that modern machine learning algorithms can accurately predict MODS recovery with a time horizon of 7 days, which would allow sufficient time for early intervention from the clinicians. As shown in Figure 3, an equally good performance was achieved when the model was trained and validated on two significantly different patient populations. For instances, patients in the SPSS is relatively younger (median age of 16.6/1.75 months in SPSS versus 42/7.7 months in LCHC in *recovery/non-recovery* episodes) with a higher PICU admission rate (78.8%/96.4% in SPSS versus 57.5%/54.8% in LCHC), while the percentage of patients with comorbidity is more prominent in the LCHC cohort than in the SPSS cohort (96.1%/99.5% in LCHC versus 34.8%/40.6% in SPSS). At an operating point of high clinical relevance (i.e. 80% recall), our model achieved a precision score over 60% in both internal and external evaluations. All the results have proven that the developed model has great potential to be applicable and generalizable in predicting MODS recovery with different clinical practices. Furthermore, when evaluating the RF model for different prediction horizons on the SPSS cohort, we noticed the performance becomes higher when the horizon is shorter, which meets our expectation that closer events are easier to predict. For the same analysis on the LCHC cohort, the validation reached high prediction performance from *d*_3_ onwards with AUROC higher than 75%, the models of *d*_3_, *d*_4_ and *d*_5_ even perform better than the *d*_6_ model we present in the study. Due to the temporal limitation of the datasets, the prediction could only be made in the first week after sepsis onset. For longer horizons, such as 2 or 3 weeks, we would expect lower prediction performance. However, sepsis in children is a highly dynamic disease with progression/recovery often seen within the first few days after admission, and the persistence of MODS on the first week is associated with high mortality, short- and long-term morbidity. Therefore, we focused on the *d*_6_ MODS recovery in this study.

Nowadays, advanced and complex machine learning models have been widely applied in healthcare for their superiority over traditional models. However, a major shortcoming is the lack of interpretability regarding the decisions from the model, as the decision-making in healthcare is a high-stake process that comes with the potential impact on the survival of the patient (Stiglic *et al.*, 2020). To make the proposed MODS recovery prediction method more understandable to the clinicians and thus provide more insights, we, therefore, used SHAP values to explain the risk factors discovered by the model. With the detailed information described in Section 4.3, the clinicians can find visual explanations of which specific features predispose the pediatric sepsis patients with MODS to recovery. Interestingly, two organ systems, namely cardiovascular and respiratory, are found to be highly critical in the early prediction of the recovery process, as most of the variables ranked in the top 10 variables are associated with these two systems. With this being found, we should pay more attention when taking care of the MODS patients with cardiovascular or respiratory failure, rather than treating them equal to patients with other types of organ system failure. In the enrichment analysis, we observed an over-representation of younger patients in false negatives and correspondingly an over-representation of older patients in the false positives, which suggests the model tends to give younger (older) children lower (higher) likelihood of recovery.

Finally, our study faced certain limitations: Despite the original SPSS cohort containing over one thousand sepsis episodes, the size of the target cohort was reduced to 256 after filtering irrelevant episodes, which is a relatively small population for data-driven machine learning approaches. Larger sample size would allow for the evaluation of prediction performance based on different risk groups stratified by age, ethnicity or chronic disorders. Another limitation we recognized in this study is that due to the retrospective nature of our observational study, we lack the possibility to compare the model’s prediction with the likelihood of MODS recovery given by a clinician, when he/she was checking the patients on bedside. Therefore, a prospective evaluation is necessary to assess the clinical utility of this MODS recovery prediction in future work.

## Ethics approval

The Swiss Pediatric Sepsis Study was approved by the ethics committees of all participating medical centers (Cantonal Ethics Committee, Inselspital, University of Bern, no. KEK-029/11). The use of data collected in Ann and Robert H. Lurie Children’s Hospital of Chicago has been approved by institutional review board (IRB) 2020-3868.

## Funding

This project has received funding from the European Union’s Horizon 2020 research and innovation program under the Marie Sklodowska-Curie grant agreement No [813533 to K.B.], and the Swiss National Science Foundation [grant number 342730_153158/1 and 320030_201060/1], the Swiss Society of Intensive Care, the Bangerter Foundation, the Vinetum and Borer Foundation, and the Foundation for the Health of Children and Adolescents.

*Conflict of Interest*: The Authors declare no conflict of interest.

*Swiss Pediatric Sepsis Study*

Principal Investigators*:* Philipp KA Agyeman, MD, Christoph Berger, MD, Eric Giannoni, MD, Martin Stocker, MD, Klara M Posfay-Barbe, MD, Ulrich Heininger, MD, Sara Bernhard-Stirnemann, MD, Anita Niederer-Loher, MD, Christian R. Kahlert, MD, Giancarlo Natalucci, MD, Christa Relly, MD, Thomas Riedel, MD, Christoph Aebi, MD, Luregn J Schlapbach, MD, FCICM for the Swiss Pediatric Sepsis Study

## Supplementary Material

btac229_Supplementary_Data
