# Supplementary material for: Prediction of recovery from multiple organ dysfunction syndrome in pediatric sepsis patients
Source: Bioinformatics. 2022 Jun 27;38(Suppl 1):i101–8. doi: 10.1093/bioinformatics/btac229 (PMC9236580; doi:10.1093/bioinformatics/btac229)
Supplement: btac229_Supplementary_Data [file btac229_supplementary_data.pdf]

Table 1: Overview of all available features and their missingness (on blood culture sampling day) in the SPSS dataset. For the MODS recovery prediction task, we employed 22 physiology features, 6 organ failure scores, 14 indicators of chronic disorder condition, and 2 patient demographic features.

	**Feature**	**% Missing SPSS**
	**Demographics**	0.0
01	Age at blood culture sampling (days)	0.0
02	Self-reported sex of patient	0.0
03	Patient is a neonate	0.0
	**Chronic condition indicators**	0.0
04	Chronic neurological disorder according to [**feudtner2014**]	0.0
05	Chronic cardiac disorder according to [**feudtner2014**]	0.0
06	Chronic lung disorder according to [**feudtner2014**]	0.0
07	Chronic urogenital disorder according to [**feudtner2014**]	0.0
08	Chronic gastrointestinal disorder according to [**feudtner2014**]	0.0
09	Chronic haematological or immunological disorder according to [**feudtner2014**]	0.0
10	Chronic metabolic disorder according to [**feudtner2014**]	0.0
11	Malformation or genetic disorder according to [**feudtner2014**]	0.0
12	Malignancy according to [**feudtner2014**]	0.0
13	Neonatal disorder according to [**feudtner2014**]	0.0
14	Surgical disorder according to [**feudtner2014**]	0.0
15	Dependence on technological assistance according to [**feudtner2014**]	0.0
16	History of transplant according to [**feudtner2014**]	0.0
17	Total number of underlying chronic disorders	0.0
18	Patient has a complex chronic condition	0.0
19	Patient was previously healthy	0.0
	**Physiology**	
20	Lowest mean arterial blood pressure (mmHg) within 24 hs	2.3
21	Lowest systolic blood pressure (mmHg) within 24 hs	2.0
22	Highest lactate (mmol/l) within 24 hs	14.1
23	Highest inspired oxygen fraction within 24 hs	25.4
24	Lowest platelet count (G/l) within 24 hs	7.8
25	Lowest white cell count (G/l) within 24 hs	9.4
26	Lowest lymphocyte cell count (G/l) within 24 hs	19.1
27	Lowest absolute (total) neutrophil cell count (G/l) within 24 hs	19.5
28	Highest total bilirubin (μmol/l) within 24 hs	37.1
29	Highest serum creatinine (μmol/l) within 24 hs	33.6
30	Lowest Glasgow Coma Scale score within 24 hs	47.7
31	Highest temperature ($^{\circ}$C) within 24 hs	3.5
32	Lowest temperature ($^{\circ}$C) within 24 hs	3.9
33	Highest heart rate (bpm) within 24 hs	1.6
34	Lowest oxygen saturation (%) within 24 hs	2.3
35	Highest respiratory rate (bpm) within 24 hs	9.0
36	Highest alanine aminotransferase (U/l) within 24 hs	45.7
37	Highest INR coagulation value within 24 hs	44.9
38	Highest white cell count (G/l) within 24 hs	7.4
39	Highest partial arterial CO_2 pressure (mmHg) within 24 hs	45.3
40	Longest central capillary refill (s) within 24 hs	48.8
41	Lowest partial arterial O_2 pressure (mmHg) within 24 hs	48.8
	**Organ scores**	0.0
42	Respiratory 2017 pSOFA score [**matics2017**]	0.0
43	Cardiovascular 2017 pSOFA score [**matics2017**]	0.0
44	Central nervous system 2017 pSOFA score [**matics2017**]	0.0
45	Renal 2017 pSOFA score [**matics2017**]	0.0
46	Hepatic 2017 pSOFA score [**matics2017**]	0.0
47	Hematological 2017 pSOFA score [**matics2017**]	0.0
48	Respiratory PELOD-2 score [**leteurtre2013**]	0.0

	Feature	% Missing SPSS
49	Cardiovascular PELOD-2 score [**leteurtre2013**]	0.0
50	Central PELOD-2 score [**leteurtre2013**]	0.0
51	Renal PELOD-2 score [**leteurtre2013**]	0.0
52	Hematological PELOD-2 score [**leteurtre2013**]	0.0
53	Respiratory failure according to the 2005 consensus [**goldstein2005**]	0.0
54	Cardiovascular failure according to the 2005 consensus [**goldstein2005**]	0.0
55	Central nervous system failure according to the 2005 consensus [**goldstein2005**]	0.0
56	Renal failure according to the 2005 consensus [**goldstein2005**]	0.0
57	Hepatic failure according to the 2005 consensus [**goldstein2005**]	0.0
58	Hematological failure according to the 2005 consensus [**goldstein2005**]	0.0

Table 2: Demographic and clinical characteristics of children in SPSS with blood culture-proven sepsis, restricted to the patients with MODS on blood culture sampling day. All 7-day episodes, $n = 256$.

**Category**	**Result SPSS**
Age at sepsis onset (months)	6.58 (0.3 - 53.5)
Preterm neonate	91 (35.5%)
Term neonate <28 days	15 (5.9%)
28–365 days	44 (17.2%)
1–4 years	48 (18.8%)
5–9 years	22 (8.6%)
10–16 years	36 (14.1%)
Female	98 (38.3%)
Male	158 (61.7%)
White European	188 (73.4%)
Asian	7 (2.7%)
African	19 (7.4%)
Other / Middle East	2 (0.8%)
mixed	21 (8.2%)
Missing values ethnicity	14 (5.5%)
Neonate	106 (41.4%)
Previously healthy child	43 (16.8%)
comorbidity	107 (41.8%)
Early-onset sepsis	24 (9.4%)
Late-onset sepsis (community-acquired)	3 (1.2%)
Late-onset sepsis (hospital acquired)	79 (30.9%)
Community-acquired sepsis	82 (32.0%)
Hospital-acquired sepsis	68 (26.6%)
Length of hospital stay after sepsis onset (days)	32 (11 - 94.3)
Coagulase-negative staphylococci	37 (14.5%)
E. coli	42 (16.4%)
Staphylococcus aureus	33 (12.9%)
Streptococcus pneumoniae	17 (6.6%)
Klebsiella pneumoniae	16 (6.2%)
Candidiasis	15 (5.9%)
Group A streptococcus	11 (4.3%)
Enterococcus	10 (3.9%)
Neisseria meningitidis	10 (3.9%)
Other Gram-positive bacteria	9 (3.5%)
Other Gram-negative bacteria	26 (10.2%)
Candidiasis	15 (5.9%)
Group A streptococcus	11 (4.3%)
Viridans group streptococci	6 (2.3%)
Pseudomonas aeruginosa	6 (2.3%)
Haemophilus influenzae	5 (2.0%)

Category	Result SPSS
Primary bloodstream	47 (18.5%)
Central line-associated bloodstream	90 (35.2%)
Urinary tract	5 (2%)
Pneumonia	30 (11.7%)
Central nervous system	21 (8.2%)
Gastrointestinal system	27 (10.5%)
Bone and joints	2 (0.8%)
Skin and soft tissue	5 (2%)
Other specific infection type	9 (3.5%)
Ear, nose, and throat	1 (0.4%)
Surgical site	7 (2.7%)
Other	9 (3.5%)
Toxic shock syndrome	7 (2.7%)
Two organ dysfunction (cumulative first 7 days)	94 (36.7%)
Three organ dysfunction (cumulative first 7 days)	74 (28.9%)
Four organ dysfunction (cumulative first 7 days)	48 (18.7%)
Five organ dysfunction (cumulative first 7 days)	28 (10.9%)
Six organ dysfunction (cumulative first 7 days)	12 (4.7%)
Not admitted to PICU	30 (11.7%)
Admitted to PICU	226 (88.3%)
Length of PICU stay after sepsis onset (days)	9 (3 - 43.75)
30-day mortality	27.0%

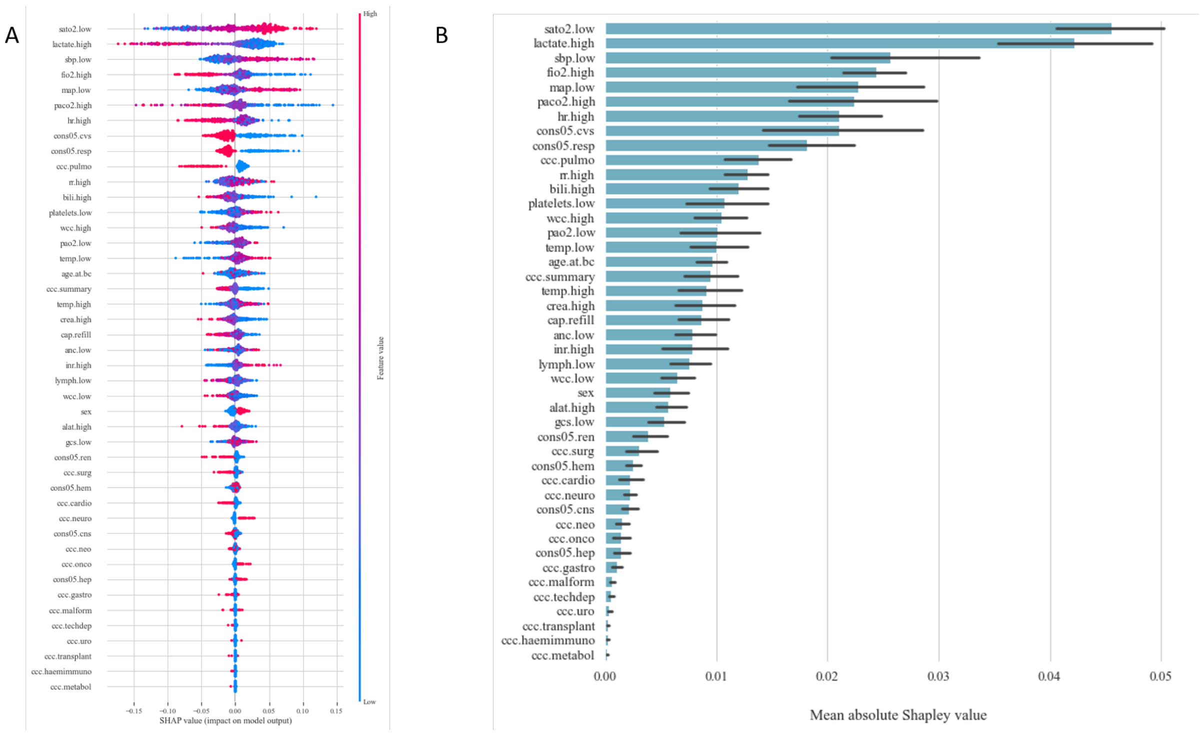

Figure 1: SHAP values with respect to the MODS recovery prediction task for all features used in the proposed model (Random Forests). **A**. SHAP values distributions. **B**. Average SHAP values.
